# Prokinetic and laxative effects of the crude methanolic extract of *Viola betonicifolia* whole plant in rodents

**DOI:** 10.1186/1472-6882-13-70

**Published:** 2013-03-27

**Authors:** Naveed Muhammad, Najeeb ur Rehman, Haroon Khan, Muhammad Saeed, Anwarul-Hassan Gilani

**Affiliations:** 1 Department of Pharmacy, University of Peshawar, Peshawar, 25120, Pakistan; 2 Natural Product Research Unit, Department of Biological and Biomedical Sciences, The Aga Khan University Medical College, Karachi, 74800, Pakistan; 3 Gandhara College of Pharmacy, Gandhara University, Peshawar, Pakistan; 4 College of Health Sciences, Mekelle University, Mekelle, Ethiopia

**Keywords:** *Viola betonicifolia*, Prokinetic, Laxative, Cholinergic, Jejunum, Ileum

## Abstract

**Background:**

The present study was aimed to provide ethnopharmacological basis for the medicinal use of *Viola betonicifolia* whole plant in indigestion and constipation.

**Methods:**

Mice were used in in-vivo prokinetic and laxative studies while in-vitro experiments were conducted on isolated tissues of rabbit and guinea-pig gut preparations suspended in a tissue bath to measure isotonic contractions.

**Results:**

The crude methanolic extract of *Viola betonicifolia* (VBME) showed partially atropine-sensitive prokinetic (50 and 100 mg/kg) and laxative (30 and 100 mg/kg) activities in mice. When tested in isolated rabbit jejunum and guinea-pig ileum, VBME caused dose-dependent contractions at 0.01-0.3 mg/mL and 0.03-5 mg/mL, respectively. The spasmogenic effect was partially sensitive to atropine, while the presence of pyrilamine, SB203186 or hexamethonium had no effect in both gut preparations. VBME partially inhibited acetylcholinesterase enzyme (19%) in the in-vitro assay. The spasmodic effect of VBME was more efficacious in guinea-pig ileum than rabbit jejunum preparation. The phytochemical analysis of the crude methanolic extract for total alkaloids and saponins revealed that the VBME is a rich source of alkaloids and saponins.

**Conclusions:**

This study showed the prokinetic and laxative effects of *Viola betonicifolia* in mice, partially mediated through cholinergic action. The in-vitro spasmodic effect of the plant extract was also partially sensitive to atropine indicating more than one mechanisms in the gut stimulant effect. This study provides a rationale for the medicinal use of *Viola betonicifolia* in indigestion and constipation.

## Background

*Viola betonicifolia* belongs to family Violaceace locally known as banafsha. It is found naturally in various countries of the world like Pakistan, India, Nepal, Srilanka, China, Malaysia and Australia. In Pakistan, it is found in the northern areas, like Swat, Hazara and district Dir. The folk use of this plant includes purgative, antipyretic, astringent, diaphoretic and anticancer. It has also been used in the treatment of various neurological disorders including epilepsy and insomnia [1]. Additionally, it has been used in the treatment of sinusitis, skin and blood disorders and pharyngitis [2]. Roots are used for kidney diseases, pneumonia and bronchitis. Flowers are recommended for the treatment of cough and colds while leaves are useful for the treatment of boils [3].

Phytochemical study revealed the presence of saponins, flavonoids, tannins, proteins, and phenolic compounds [4-6]. The plant has been reported to possess multiple biological activities, such as nematicidal, antioxidant, larvicidal, and cytotoxic [4], antipyretic, analgesic and anti-inflammatory [5], and neuroprotective [6]; however, plant has not been studied for its effectiveness in gut disorders. The current study was designed to provide scientific evidence to the ethnobotanical uses of the plant in the treatment of constipation and indigestion using various pharmacological models.

## Methods

### Preparation of the crude extract

Whole plant (aerial part plus roots) of *V. betonicifolia* was collected from Swat, Khyber Pakhtunkhwa, Pakistan, in April 2010. Plant specimen was identified by Taxonomist, Department of Botany, University of Peshawar and a specimen was deposited there in the herbarium with voucher number 6410/Bot. The collected whole plant (12 kg) was air dried and powdered. The powder was extracted by maceration with methanol at room temperature for 14 days with occasional shaking. The methanolic extract was filtered and concentrated under vacuum using rotary evaporator at low temperature (45°C) to a thick and dark brown crude extract (VBME). The approximate yield was 22% w/w. The methanolic extract was completely soluble in normal saline (0.9% w/v) and distilled water for the in-vivo and in-vitro experiments, respectively.

### Chemical and reagents

Acetylcholine perchlorate (ACh), atropine sulphate, carbamylcholine or carbachol (CCh), histamine hydrochloride, 5-hydroxytryptamine (5-HT), pyrilamine maleate and hexamethonium chloride were purchased from Sigma-Aldrich Chemicals Company (St Louis, MO, USA). SB203186 (1-piperidinylethyl-1H-indole-3-carboxylate) was purchased from Tocris (Ballwin, MO, USA). Chemicals used for making physiological salt solutions including potassium chloride, calcium chloride, glucose, magnesium chloride, magnesium sulfate, potassium dihydrogen phosphate, sodium bicarbonate, sodium dihydrogen phosphate and sodium chloride were obtained from Merck (Darmstadt, Germany). All chemicals used were of the analytical grade available and solubilized in distilled water.

### Animals

BALB/c mice (weighing 20–25 g, n = 96) were used for the in-vivo prokinetic and laxative studies in such a way that both activities comprised of eight groups of mice (6 animals in each), while in-vitro experiments were conducted on gut preparations from guinea-pigs (weighing 400–600 g, n = 5) and local bred rabbits (weighing 1–1.5 kg, n = 7) of either sex, housed at the animal house of the Aga Khan University under the controlled environment (23–25°C). The animals were kept in their respective cages with sawdust (changed after every 48 h) and were fasted according to the protocols of the study. In routine, they were given tap water *ad libitum* and a standard diet consisting of (g/kg): flour 380, fiber 380, molasses 12, NaCl 5.8, nutrivet L 2.5, potassium metabisulfate 1.2, vegetable oil 38, fish meal 170 and powdered milk 150. The experimental protocols were approved by the ethical committee of the Department of Pharmacy, University of Peshawar, Peshawar, Pakistan. All the experiments were performed in compliance with the rulings of the Institute of Laboratory Animal Resources, Commission on Life Sciences, National Research Council [7].

### Phytochemical screening

The saponin contents in the crude extract were determined according to documented method [8]. Briefly, 2 g of test samples were taken in a beaker and 50 mL of petroleum ether was added and heated gently on water-bath to 40°C for 5 min with regular shaking. The petroleum ether was filtered and repeated the operation twice with further 50 mL of petroleum ether. The marc obtained was extracted with 4 × 60 mL of methanol on gentle heating. The methanol layer was concentrated to approximately 25 mL on water-bath and 150 mL of dry acetone was added to precipitate the saponins, which was followed by filtration and drying in oven at 100°C for constant weight. For quantification of total alkaloid contents the marc obtained was acidified with 100 mL of 20% acetic acid in ethanol and allowed to extract for 4 h. The resulting solution was filtered, concentrated and then basified with concentrated ammonium hydroxide to pH 9 followed by precipitation. The final weight of precipitated mass was designated as the total alkaloid contents.

### In-vivo experiments

#### Charcoal meal GI transit test

The mice were divided into various groups (*n* = 6). The group treated with saline (10 mL/kg) was served as a negative control, while the group treated with CCh (1 mg/ kg) served as positive control. The remaining groups were treated with VBME (50 and 100 mg/kg, orally) and atropine (10 mg/kg, intraperitoneally). After 15 min of treatment, each animal received 0.3 mL of charcoal meal in the form of suspension in distilled water containing 10% gum acacia and 10% vegetable charcoal. After 30 min of the above treatments, animals were sacrificed by cervical dislocation and the whole small intestine was removed. The distance travelled by charcoal was measured and the percent movement was calculated. In order to assess the involvement of acetylcholine like prokinetic effect of the extract and CCh, some groups were treated with atropine (10 mg/kg, i.p.) 15 min prior to the administration of the VBME or CCh [9].

#### Laxative activity test

In accordance with the previous method [10], mice fasted for 6 h before the experiment were placed individually in cages lined with clean filter paper. The animals were divided into eight groups (n = 6); the first group acted as the negative control and received saline (10 mL/kg, p.o.) and the second group received atropine (10 mg/kg, i.p.) only. The third group received CCh (1 mg/kg, p.o), which served as the positive control. The fourth and fifth groups received oral doses of 30 and 100 mg/kg of VBME, respectively. To determine the mechanism underlying of its laxative effect, separate sets of mice (group # 6, 7 and 8) were pretreated with atropine (10 mg/kg, i.p.) one h before administration of the extract or CCh. After 18 h, the feces production (total number of feces and total number of wet feces per group) in all animals was counted, and the % increase in wet feces relative to that of total fecal output was recorded, which was considered as the laxative effect [11].

#### In-vitro experiments

By following the previously described methods [12], isolated gut preparations of rabbit jejunum and guinea-pig ileum were obtained subsequent to cervical dislocation of the animals; the abdomen was cut opened, required tissues (jejunum and ileum) were excised and transferred to a petri dish containing normal Tyrode’s solution continuously aerated with carbogen (a mixture of 5% carbon dioxide and 95% oxygen). The adjacent mesenteric connections of preparations were cut and cleaned off. Tissue preparations jejunum or ileum of 2–3 cm long were mounted in 10 mL tissue baths containing Tyrode’s solution maintained at 37°C and aerated with carbogen. The composition of Tyrode’s solution (mM) was KCl 2.68, NaCl 136.9, MgCl_2_ 1.05, NaHCO_3_ 11.90, NaH_2_PO_4_ 0.42, CaCl_2_ 1.8, and glucose 5.55 (pH 7.4). A preload of one g was applied to each tissue, and the contractile responses were recorded using isotonic transducer 50–6360 (Harvard Apparatus, Holliston, MA, USA) coupled with either a student oscillograph (Harvard Apparatus) or PowerLab (ML-845) data acquisition system (AD Instruments; Sydney, Australia) and a computer using chart software (version 5.3). The tissues were allowed to equilibrate for a period of 30 min, and then stabilized with sub-maximal concentration of acetylcholine (ACh, 0.3 μM). The tissues were presumed stable only after the reproducibility of the said responses. The VBME was examined later for any spasmodic activity on jejunum and ileum preparations at concentrations ranged from 0.01 to 5.0 mg/mL.

#### Acetylcholinesterase inhibition assay

The aceylcholinesterase (AChE) inhibitory activity was measured spectrophotometrically using method of Ellman [13]. For this purpose, an enzyme kit (DACE-100-QuantiChrom™ Acetylcholinesterase Assay Kit) based on the Ellman method was employed. Electric-eel AChE (type VI-S) was used, while acetylthiocholine was used as substrates of the reaction. 5,5-dithiobis[2-nitrobenzoic acid] (DTNB) was used for the measurement of cholinesterase activity. 140 ml of the 100 mM sodium phosphate buffer (pH 8.0), 10 mL of DTNB (1 mmol/L), 20 mL of the crude extract solution and 20 ml of AChE/BChE (0.05 mg/ml of AChE and 0.2 mg/ml of BChE) solution were mixed and incubated for 15 min at 25°C. The reaction was then initiated by the addition of 10 ml of acetylthiocholine (0.71 mM of acetylthiocholine). The hydrolysis of acetylthiocholine was monitored by the formation of yellow 5-thio-2-nitrobenzoate anion as the result of the reaction of DTNB with thiocholine, released by the enzymatic hydrolysis of acetylthiocholine at a wavelength of 412 nm using a spectrophotometer (Beckman DU-600 spectrophotometer, USA) in the quartz cuvettes (Starna, U.K., no: 1 OG 5391). The extract and the control were dissolved in EtOH (5%). The rate of enzymatic reaction was finally determined by Ellman equation:

$$\mathrm{Rate}\left(\mathrm{mols}\;/\;L\;/\min\right)=\mathrm{Change}\;\mathrm{in}\;\mathrm{absorbance}\;\mathrm{per}\;\min/13,600$$

All the inhibition studies were conducted in 96-well micro-titer plates using Spectra Max 340 (Molecular Devices, CA, USA).

#### Statistical analysis

All the data expressed are expressed as mean ± standard error of mean (S.E.M., n = number of experiments) and the median effective concentrations (EC_50_ values) with 95% confidence intervals (CI). One way analysis of variance (ANOVA) followed by Dunnett’s test or unpaired *t-*test was used to assess the laxative activity, while one-way ANOVA followed by Tukey’s test was employed for the effect of plant extract in charcoal meal transit. The concentration-response curves (CRCs) were analyzed by non-linear regression and two-way ANOVA followed by Bonferroni’s post-test correction or unpaired *t*-test was used for multiple comparisons of CRCs with the respective control. All the graphing, calculations and statistical analysis were performed using GraphPad Prism 4 for windows (GraphPad Software, San Diego, California, USA).

## Results

### Phytochemical screening

The crude methanolic extract was found a rich source of alkaloid (7.45 mg/g) and saponins contents (7.23 mg/g).

### In-vivo findings

#### Effect of VBME on charcoal meal intestinal transit

**Figure 1:**
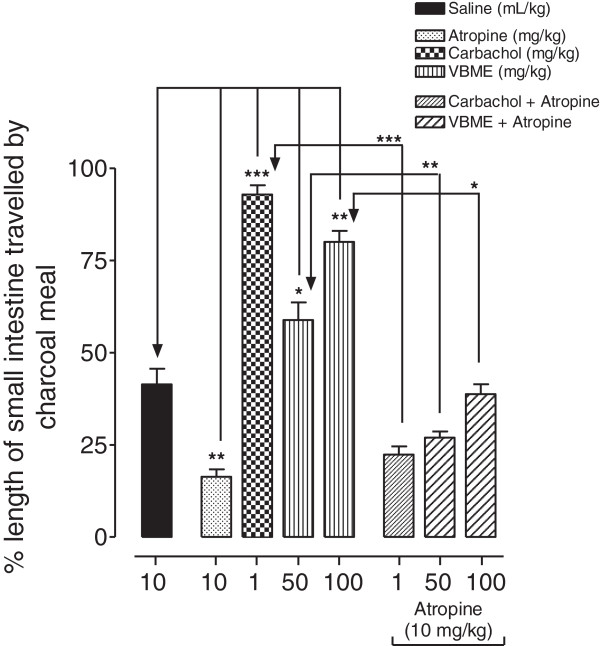
**Bar diagram showing the dose-dependent effect of crude methanolic extract of *Viola betonicifolia *(VBME) on the travel of charcoal meal through small intestine of mice, in the absence and presence of atropine.** One-way ANOVA followed by Tukey’s test. *P < 0.05, **P < 0.01 and ***P < 0.001.

VBME exhibited a dose-dependent increase in the propulsive movement of charcoal meal as shown in Figure 1. The percent movement of charcoal meal through the small intestine after treatment with VBME was 58.33 and 79.66 at the test doses of 50 and 100 mg/kg respectively, while in saline, atropine and CCh treated groups the charcoal meal travelled 51.66, 16.3 and 92.67% respectively. When VBME and CCh were restudied for their influence on transit of charcoal meal in mice pretreated with the intraperitoneal injection of atropine, all the excitatory effects were markedly reduced and no abnormal behavior was observed in animals even afte four hours of injection.

#### Laxative activity

VBME treatment produced 61.16 ± 2.18% and 86.83 ± 4.8% (n = 6) wet feces in mice at 30 and 100 mg/kg, respectively. The positive control, CCh (1 mg/kg) produced 88.16 ± 3.07% wet feces, while the saline and atropine treated groups did not form any wet feces. When VBME (30 and 100 mg/kg) was studied for its positive influence on wet feces in mice pretreated with atropine, the effect declined to 36.6 ± 5.2% and 46.3 ± 11.7%, respectively; further details are shown in Table 1.

**Table 1:** **Effect of atropine on the laxative activity of the crude methanolic extract of*Viola betonicifolia*(VBME) in mice**

**Group no.**	**Treatment**	**Dose (mg/kg)**	**Defecation/group**	**Number of wet feces/group**	**% of wet feces**
1	Saline (p.o mL/kg)	10	3 ± 0.36	0	0
2	Atropine (i.p)	10	1 ± 0.63	0	0
3	Carbachol (p.o)	1	11.5 ± 0.5**	10.0 ± 0.54***	88.16 ± 3.07
4	VBME (p.o)	30	8.3 ± 0.88**	5.1 ± 0.9*	61.16 ± 2.18
5		100	11.1 ± 1.3**	9.5 ± 71**	86.83 ± 4.8
6	Carbachol + Atropine (i.p)	1 + 10	4 ± 0.5**	0.83 ± 0.3***	22.5 ± 10.1
7	VBME (p.o) + Atropine (i.p.)	30 + 10	5.3 ± 0.9*	1.83 ± 0.3***	36.6 ± 5.2
8		100 + 10	4.6 ± 0.6**	2 ± 0.36***	46.3 ± 11.7

Values shown are mean ± S.E.M, n = 6. *P < 0.05, **P < 0.01 and ***P < 0.001 show a comparison of group # 2,3,4 and 5 vs. group # 1 (One-way ANOVA followed by Dunnett’s test), group # 5 vs. group # 2, group # 6 vs. group # 3 and group # 7 vs. group # 4 (unpaired *t-* test).

#### In-vitro findings

##### Effect of VBME on rabbit and guinea-pig gut preparations

**Figure 2:**
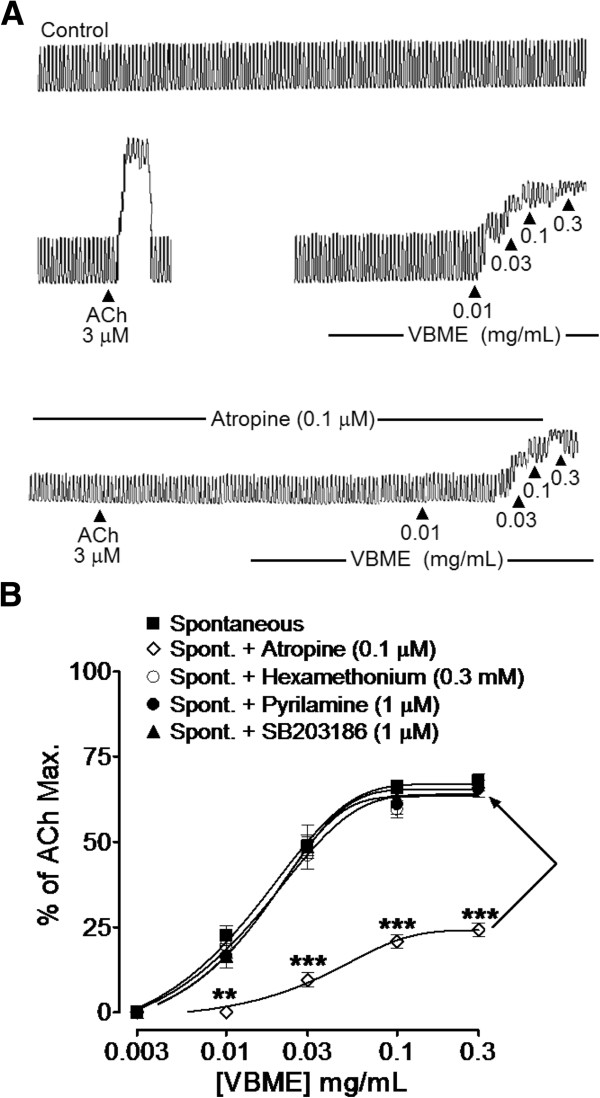
**Typical tracing (A) showing the effect of the crude methanolic extract of*Viola betonicifolia*(VBME) and acetylcholine (ACh) in the absence and presence of atropine (0.1 μM) and (B) showing the dose–response curves of the spasmogenic effect of the VBME in the absence and presence of atropine (0.1 μM), pyrilamine (1 μM), hexamethonium (0.3 mM) or SB203186 (1 μM), in isolated rabbit jejunum preparations.** The values shown are mean ± S.E.M of 4–7 individual experiments and expressed as the percentage of ACh maximum response. *P < 0.05, **P < 0.01 and ***P < 0.001 (Two-way ANOVA, followed by bonferoni post-test correction or unpaired *t*-test).

In spontaneously contracting rabbit jejunum preparations, VBME caused a concentration-dependent stimulant effect at 0.01-0.3 mg/mL (Figure 2A), reaching to its maximum effect of 67.33 ± 2.67% (mean ± S.E.M; n = 5) of ACh maximum (Figure 2B). Pretreatment of the tissue with atropine (0.1 μM) significantly (p < 0.001) blocked the spasmogenic effect of VBME with remaining maximum spasmodic effect declined to 23.33 ± 2.40% (mean ± S.E.M; n = 4), while the presence of hexamethonium, pyrilamine or SB203186 did not alter (*P* > 0.05) its effect (Figure 2A and B).

**Figure 3:**
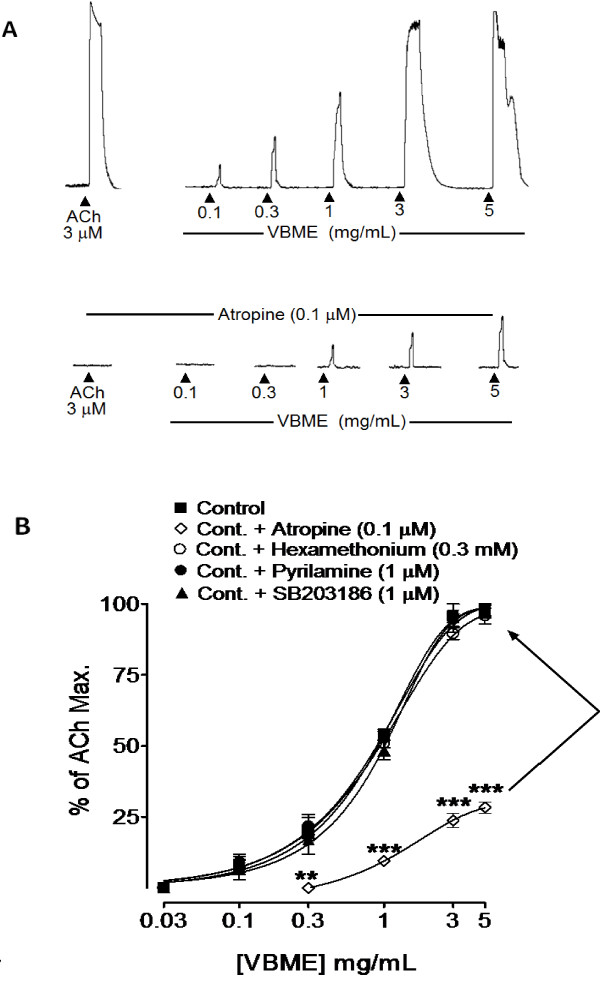
**Typical tracing (A) showing the partial atropine-sensitive spasmogenic effect of the crude methanolic extract of*Viola betonicifolia*(VBME) in comparison to acetylcholine (ACh) in the absence and presence of atropine (0.1 μM) and (B) showing the dose–response curves of the spasmogenic effect of the VBME in the absence and presence of atropine (0.1 μM), pyrilamine (1 μM), hexamethonium (0.3 mM) or SB203186 (1 μM), in isolated guinea-pig ileum preparations.** The values shown are mean ± S.E.M of 4–7 individual experiments and expressed as the percentage of ACh maximum response. *P < 0.05, **P < 0.01 and ***P < 0.001 (Two-way ANOVA, followed by bonferoni post-test correction or unpaired *t*-test).

When tested in guinea-pig ileum, VBME (0.03-5 mg/mL) exhibited a strong spasmodic effect in this quiescent preparation (Figure 3A), reaching its highest 96.66 ± 3.33% (mean ± S.E.M; n = 5), close the ACh maximum (Figure 3B). The efficacy of the contractile effect of VBME in guinea-pig ileum was found higher (p < 0.05) than observed in rabbit jejunum preparations. When the spasmogenic effect of VBME was redetermined in the presence of different antagonists, it was partially blocked in the presence of atropine with remaining maximum spasmodic effect of 28.66 ± 2.90% (mean ± S.E.M; n = 5), while the effect remained unchanged (p > 0.05) in the presence of hexamethonium, pyrilamine or SB203186 (Figure 3A and B), like that observed in rabbit jejunum.

#### Effect on acetylcholinesterase inhibition

A weak acetylcholinesterase enzyme inhibition (19%) was demonstrated by VBME, in the in-vitro assay.

## Discussion

Keeping in view the medicinal use of *Viola betonicifolia* in gut disorders, such as indigestion and constipation, its crude extract (VBME) was tested in mice, where it propelled charcoal meal through the small intestine and increased the production of wet feces, hence showing prokinetic and laxative activities, similar to the effect of carbachol, a standard cholinergic agonist and accelerator of intestinal contents (Brown and Taylor, 2006). These gut stimulatory actions of the extract were found partially sensitive to atropine, a muscarinic receptor blocker [14], indicating the presence of some ACh-like component(s) in addition to other gut stimulant constituent(s). ACh is a neurotransmitter of the parasympathetic nervous system and is known to cause gastrointestinal stimulation through the activation of muscarinic receptors [15], hence, the presence of ACh-like constituents partly explains its medicinal use in constipation and as digestive aid. To further study the possible mode of the observed prokinetic and laxative properties of the extract, isolated ileum preparations of mouse was used. But VBME did not show any stimulant effect on the baseline contraction (data not shown) of this tiny preparation. Hence, whentested in isolated rabbit jejunum and guinea-pig ileum, considered suitable preparations to study effects on gut motility [16], VBME showed a dose-dependent gut stimulant effect, partially sensitive to atropine in both preparations, whereas, the comparable stimulatory effect of acetylcholine was completely inhibited in the atropine pretreated preparations. Partial atropine-sensitive gut stimulatory effect of VBME was also supported by acetylcholinesterase inhibition assay, thus, showing a common mechanism of muscarinic receptor activation along with some additional spasmodic component(s). However, the efficacy of gut stimulant effect was high in guinea-pig ileum than that found in rabbit jejunum. To further study the nature of the unknown additional spasmodic components, other than cholinergic, the stimulant effect was restudied in the presence of pyrilamine, a histaminic type-1 (H_1_) receptor blocker [17], hexamethonium, a ganglion blocker [18] or SB203186, a serotonergic (5-HT) receptor antagonist [19]. But VBME showed complete resistance to these antagonists, clearly suggesting some additional mechanism(s), independent of histamine, nicotine or 5-Hydroxytryptamine (5-HT, serotonin) receptors activation. Other mechanisms known for their gut stimulant property, which have not been ruled out in this study include certain prostaglandins [20], platelet activating factor [21], nitric-oxide-donating or releasing compounds [22], dopaminergic antagonists [23], cholecystokinin [23] and tachykinins [24].

Recently we have reported the anthelmintic and nematicidal properties of the VBME and its subsequent solvent fractions [4,25]. It is very interesting to note that VBME possesses combination of anthelmintic and laxative activities which has the merit because the laxative and prokinetic properties of the tested extract will be helpful in the expulsion of worms or nematodes from gut. Regarding the preliminary phytochemical study, the VBME is known to be a rich source of alkaloids, flavonoids, phenolic compounds and saponins [5], while in case of quantitative phytochemical profile of the plant, it is clear from results of this study that VBME is a rich source of alkaloids and saponins. We have recently reported the quantification of flavonoids and phenolic contents in various solvent fractions of this plant. The total polyphenols were of higher in quantity in methanolic extract as compared to water fraction [4]. The presence of alkaloids and saponins as the plant constituents, which are known to possess gut stimulatory properties [26,27], may explain the gut stimulant actions of the plant extract, though further studies are required to know the specific chemical(s) responsible for the tested biological activities.

The screening of methanolic extract instead of aqueous fraction for this study was due to the presence of polyphenols in the methanolic extract which are mostly responsible for such type of activities. More useful explanation for not using the aqueous extract can be ascribed to the presence of enzyme polyphenol oxidase, which degrades polyphenols in water extracts, whereas in methanol they are inactive. Moreover, water is a better medium for the occurrence of the micro-organisms as compared to methanol [28]. Our research group is working on isolation of active constituents from this plant through column chromatography and we intend to make use of HPLC at later stage and hopefully our next publication will contain HPLC data for the isolation of active principles from this plant.

## Conclusion

This study shows that the prokinetic, laxative and spasmodic activities of *Viola betonicifolia* are partially mediated through cholinergic action along with some unknown additional mechanism(s), and the pattern of activity was confirmed in the *in-vitro* experiments using rabbit jejunum and guinea-pig ileum preparations. Thus, this study provides sound mechanistic basis for the medicinal use of *Viola betonicifolia* in gastrointestinal disorders, such as indigestion and constipation.

## Abbreviations

VBME: *Viola betonicifolia* methanolic extract; ACh: Acetylcholine; CCh: Carbamylcholine/Carbachol; 5-HT: 5-hydroxytryptamine; i.p: Intraperitonial; p.o: Per oral; GI: Gastrointestinal; CRCs: Concentration-response curves; S.E.M: Standard error of means.

## Competing interests

The authors declare that they have no conflicts of interest.

## Authors’ contributions

NM and NR: Performed experimental work. HK: Manuscript writing. MS: Project supervisor. AHG: Project supervisor and technical expertise. All authors read and approved the final manuscript.

## Pre-publication history

The pre-publication history for this paper can be accessed here:

http://www.biomedcentral.com/1472-6882/13/70/prepub
